# Retraction: MicroRNA-302c enhances the chemosensitivity of human glioma cells to temozolomide by suppressing P-gp expression

**DOI:** 10.1042/BSR-2019-0421_RET

**Published:** 2022-12-22

**Keywords:** Human glioma, MicroRNA-302c, P-pg, Temozolomide, Temozolomide

This article is being retracted from *Bioscience Reports* at the request of the authors following receipt of a notification from a reader, alerting the Editorial Office to various concerns within the data and figures. Suspected similarities have been identified between the miR-302c mimics panel of Figure 3A and the Figure 3A HCT116 miR-208a-3p inhibitor panel of Wu et al. 2019 (doi: 10.1042/bsr20181598), and between the Figure 2G mimics NC panel and the Figure 2D HCT116 Inhibitor NC panel of the same paper by Wu et al. 2019 (doi: 10.1042/bsr20181598). The authors are unable to correct the article and wish to retract the article. The Editor-in-Chief and Editorial Board agree with the retraction.

